# SCUBE3 serves as an independent poor prognostic factor in breast cancer

**DOI:** 10.1186/s12935-021-01947-3

**Published:** 2021-05-18

**Authors:** Qin Huo, Xi He, Zhenwei Li, Fan Yang, Shengnan He, Ling Shao, Ye Hu, Siqi Chen, Ni Xie

**Affiliations:** 1 Biobank, Institute of Translational Medicine, Shenzhen Second People’s Hospital, First Affiliated Hospital of Shenzhen University, Shenzhen, 518035 China; 2 The Eighth Affiliated Hospital, Sun Yat-Sen University, Shenzhen, 518033 China

**Keywords:** SCUBE3, Expression, Breast cancer, Prognostic factor, Cox regression analysis

## Abstract

**Background:**

Accumulating evidences indicate that the signal peptide-CUB-EGF-like domain-containing protein 3 (SCUBE3) plays a key role in the development and progression of many human cancers. However, the underlying mechanism and prognosis value of SCUBE3 in breast cancer are still unclear.

**Methods:**

The clinical data of 137 patients with breast cancer who underwent surgical resection in Taizhou Hospital of Zhejiang Province were retrospectively analyzed. We first conducted a comprehensive study on the expression pattern of SCUBE3 using the Tumor Immune Estimation Resource (TIMER) and UALCAN databases. In addition, the expression of SCUBE3 in breast tumor tissues was confirmed by immunohistochemistry. The protein–protein interaction analysis and functional enrichment analysis of SCUBE3 were analyzed using the STRING and Enrichr databases. Moreover, tissue microarray (TMA) was used to analyze the relationship between SCUBE3 expression levels and clinical-pathological parameters, such as histological type, grade, the status of estrogen receptor (ER), progesterone receptor (PR), and human epidermal growth factor receptor (HER2). We further supplemented and identified the above results using the UALCAN and bc-GenExMiner v4.4 databases from TCGA data. The correlation between the expression of SCUBE3 and survival was calculated by multivariate Cox regression analysis to investigate whether SCUBE3 expression may be an independent prognostic factor of breast cancer.

**Results:**

We found that the expression level of SCUBE3 was significantly upregulated in breast cancer tissue compared with adjacent normal tissues. The results showed that the distribution of breast cancer patients in the high expression group and the low expression group was significantly different in ER, PR, HER2, E-cadherin, and survival state (p < 0.05), but there was no significant difference in histologic grade, histologic type, tumor size, lymph node metastasis, TMN stage, subtypes, or recurrence (p > 0.05). In addition, the high expression of SCUBE3 was associated with relatively poor prognosis of ER- (p = 0.012), PR- (p = 0.029), HER2 + (p = 0.007). The multivariate Cox regression analysis showed that the hazard ratio (HR) was 2.80 (95% CI 1.20–6.51, p = 0.0168) in individuals with high SCUBE3 expression, and HR was increased by 1.86 (95% CI 1.06–3.25, p = 0.0300) for per 1-point increase of SCUBE3 expression.

**Conclusions:**

These findings demonstrate that the high expression of SCUBE3 indicates poor prognosis in breast cancer. SCUBE3 expression may serve as a potential diagnostic indicator of breast cancer.

**Supplementary Information:**

The online version contains supplementary material available at 10.1186/s12935-021-01947-3.

## Introduction

Breast cancer is the most common malignancy tumor and the leading cause of cancer death in women [1, 2]. Although about 60% of breast cancer patients can receive chemotherapy alone or in combination with other targeted therapies [3], a meta-analysis of 123 randomized trials showed that chemotherapy can only reduce recurrence and mortality by 20–33% [4]. Therefore, finding new diagnostic biomarkers and therapeutic targets will benefit more breast cancer patients.

Signal peptide-CUB-EGF-like domain-containing protein 3 (SCUBE3) is a member of the SCUBE family and is related to murine embryogenesis and development [5]. It is a kind of secretory cell surface glycoprotein first recognized in human umbilical vein endothelial cells [6]. The SCUBE3 encodes a 993 amino acid polypeptide, and its protein domain structure consists of at least five motifs: an N-terminal signal peptide, followed by an amino acid copy of an EGF (epidermal growth factor) like repeat, a spacer region, three cysteine-rich domains, and a complement protein C1r / C1s, Uegf, and Bmp1 (CUB) C-terminal domain [7]. Wu B T et al. revealed that SCUBE3 was highly expressed in primary osteoblasts tissues and cells, but low in cardiac tissues and vascular cells [8]. The extracellular SCUBE3 protein may play an important role in regulating the bioavailability of transforming growth factor-β (TGF-β) [9]. In addition, the expression of SCUBE3 has been proved to be closely related to the prognosis of patients. [10]. Accumulating studies have shown that SCUBE3 is closely related to the invasion and metastasis of lung cancer. It mainly triggers the TGF-β pathway, and then promotes epithelial-mesenchymal transition (EMT) [11, 12] and tumor angiogenesis [13]. More recently, the survival time of NSCLC patients with high expression of SCUBE3 was significantly shorter than that of NSCLC patients with low expression of SCUBE3, which supports the expression of SCUBE3 as an independent prognostic factor of NSCLC [14]. However, it is unclear whether and how SCUBE3 promotes breast cancer progression. Therefore, understanding the regulation and molecular function of SCUBE3 may identify potential targets for the diagnosis and treatment of breast cancer.

In this study, we performed tissue microarray (TMA) studies on 137 patients with breast cancer. We studied the expression pattern of SCUBE3 using the Tumor Immune Estimation Resource (TIMER) and UALCAN databases, and verified the results by immunohistochemical staining. The association between SCUBE3 expression level and tumor prognosis was analyzed using clinical data of these patients and Kaplan–Meier analysis. The protein–protein interaction analysis and functional enrichment analysis of SCUBE3 were analyzed using the STRING and Enrichr databases. We performed multivariate Cox regression analysis to investigate whether SCUBE3 expression may be an independent prognostic factor of breast cancer. We hypothesized that the possible oncogenic activity of SCUBE3 may impact the prognosis of breast cancer patients. Our findings in this study will help us enhance the understanding of the potentially positive role of SCUBE3 in breast cancer, and provide a theoretical basis for the early diagnosis, prognostic, and targeted therapy of breast cancer.

## Methods and materials

### Tissue specimen selection

This retrospective study was performed on the archived tissues of 140 diagnosed cases of breast cancer patients who underwent surgical resection in Taizhou Hospital (Zhejiang, China) from January 2005 to September 2012. None of these 140 patients received chemotherapy or radiation therapy before surgery. The paraffin block of each case was reviewed by the authors and experienced pathologists based on the presence of sufficient tissue in the block for TMA studies. This study was approved by the Ethics Committee of Taizhou Hospital (Zhejiang, China) in accordance with the principles of the Declaration of Helsinki. One hundred and forty paraffin-embedded TMA blocks were prepared and analyzed by the National Engineering Center for Biochip at Shanghai (TMA, HBreD140Su03). One patient was missed during the follow-up period, two tissue blocks were lost during the experimental procedure, and these three samples were excluded from this study. For the remaining 137 patients included in this study, distribution of SCUBE3 expression with clinicopathologic characteristics of breast cancer was listed in Table 1. In this study, according to the 2019 Chinese society of Clinical Oncology breast cancer diagnosis and treatment guidelines, we determined that the molecular typing method is: Ki-67 < 15% is low expression, Ki-67 > 30% is high expression. Therefore, our operational definitions for molecular typing method is: luminal A (ER + /PR + / HER2-, Ki67 low expression), luminal B (HER2-, ER + /HER2-, Ki67 high expression or PR-), HER-2 overexpression (ER-/PR-/HER2 +); In ER + /PR-/HER2 + and triple negative (ER-/PR-/ HER2-), Ki-67 < 15% was low expression and Ki-67 > 30% was high expression.

**Table 1:** Distribution of SCUBE3 expression with clinicopathologic characteristics of breast cancer (N = 137)

Variable		SCUBE3 expression		p-value
	Total	Low	High	
Number	137	106 (77.4%)	31 (22.6%)	
Age, years	55.9 ± 11.7	55.9 ± 11.8	55.7 ± 11.7	0.952
Histologic grade				0.514
2	95 (69.3%)	81 (85.3%)	14 (14.7%)	
3	42 (30.7%)	25 (59.5%)	17 (40.5%)	
Histologic type				0.399
Invasive ductal	114 (83.2%)	86 (75.4%)	28 (24.6%)	
Other	23 (16.8%)	20 (87.0%)	3 (13.0%)	
Tumor size, cm				0.0798
≤ 2	55 (40.1%)	42 (76.4%)	13 (23.6%)	
> 2	82 (59.9%)	64 (78.0%)	18 (22.0%)	
Lymph node metastasis				0.907
Negative	72 (52.6%)	55 (76.4%)	17 (23.6%)	
Positive	65 (47.4%)	51 (78.5%)	14 (21.5%)	
TMN stage				0.0725
I	39 (28.5%)	30 (76.9%)	9 (23.1%)	
II	52 (38.0%)	41 (78.8%)	11 (21.2%)	
III	46 (33.6%)	35 (76.1%)	11 (23.9%)	
ER				0.018
Negative	31 (23.1%)	19 (61.3%)	12 (38.7%)	
Positive	103 (76.9%)	55 (53.4%)	48 (46.6%)	
Missing	3	2	1	
PR				0.015
Negative	24 (17.8%)	15 (62.5%)	9 (37.5%)	
Positive	111 (82.2%)	60 (54.1%)	51 (45.9%)	
Missing	2	1	1	
HER2				0.039
Negative	109 (87.9%)	64 (58.7%)	45 (41.3%)	
Positive	15 (12.1%)	9 (60.0%)	6 (40.0%)	
Missing	13	11	2	
E-cadherin				0.020
Negative	45 (38.1%)	30 (66.7%)	15 (33.3%)	
Positive	73 (61.9%)	62 (84.9%)	11 (15.1%)	
Missing Subtypes	19	14	5	0.706
Luminal A	79 (62.2%)	35 (44.3%)	44 (55.7%)	
Luminal B	17 (13.4%)	9 (53.0%)	8 (47.1%)	
HER2	16 (12.6%)	6 (37.5%)	10 (62.5%)	
TNBC	25 (19.7%)	10 (40%)	15 (60.0%)	
Missing	10	3	7	
Recurrence				0.855
Yes	38 (27.7%)	29 (76.3%)	9 (23.7%)	
No	99 (72.3%)	77 (77.8%)	22 (22.2%)	
Survival state				0.032
Survival	108 (78.8%)	89 (82.4%)	19 (17.6%)	
Death	29 (21.2%)	12 (41.4%)	17 (58.6%)	

*ER* estrogen receptor, *PR* progesterone receptor, *HER2* human epidermal growth factor receptor; TMN stage was according to the seventh edition of the Guidelines for the American Journal of Critical Care. Variables are expressed as n (%) or the mean ± SD

### TIMER database analysis for SCUBE3 mRNA expression in breast cancer tissue

The transcription levels of SCUBE3 in different cancers were analyzed using TIMER (https://cistrome.shinyapps.io/timer/) [15], in which different cancer types (tumor/normal) were plotted on the x-axis and SCUBE3 expression was plotted on the y-axis, and the gene expression level was presented as log2-RSEM.

### Immunohistochemistry

TMA slides were deparaffinized and rehydrated through graded alcohols and then subjected to an antigen retrieval procedure (10 mM sodium citrate, 0.05% Tween-20, pH 6.0 for 25 min). Endogenous peroxidase was blocked with 0.3% hydrogen peroxide for 10 min at room temperature. After incubation with the primary rabbit polyclonal anti-SCUBE3 antibody (ab189955, 1:200 dilution, Abcam, Cambridge, UK) overnight at 4 °C, immunoreactivity was developed with an Envision System (DakoCytomation, Glostrup, Denmark) with diaminobenzidine as chromogen. The slides were finally counterstained with hematoxylin (Zymed Laboratories Inc., San Francisco, CA, USA). Incubation with a normal rabbit IgG instead of the primary antibody was not immunoreactive (data not was shown), supporting a good specificity anti-SCUBE3 antibody. Besides, we defined an appropriate scoring system based on the immune response scoring method proposed by Remmele and Stegner, using the product of staining intensity and the percentage of positive cells. The staining intensity is divided into 4 levels, that is, level 0 is negative if no positive cells are seen, level 1 is weakly positive, level 2 is moderately positive, and level 3 is strong positive. The percentage of positive cells is divided into 5 levels, that is, level 0 is negative, level 1 ≤ 10%, level 2 is 11%–50%, level 3 is 51%–80%, and level 4 > 80%. When the product of the staining intensity and the percentage of positive cells > 3 points, it is considered an immune response ( +) [16]. The expression density of SCUBE3 in breast cancer tissue was quantitated by scoring staining intensity, including negative (–), weak ( +), moderate (+ +), and strong (+ + +) staining, respectively [17].

### Protein–protein interaction analysis and functional enrichment analysis for SCUBE3

The STRING database (https://string-db.org/) [18] that searches for interactions between known proteins and predictive proteins was used to analyze SCUBE3 and homo sapiens. Protein name: SCUBE3, and organism: homo sapiens were analyzed. Enrichr, a comprehensive tool for gene enrichment analysis (http://amp.pharm.mssm.edu/Enrichr) [19], was searched to analyze the genes interacting with SCUBE3, and gene ontology (GO) enrichment analysis was further performed to identify gene interactions from three functional categories, i.e., biological process, molecular function, and cellular component [20].

### Evaluation of clinical and histopathological parameters

Clinical and pathological parameters, such as gender, age, histological type, grade, tumor size, the status of lymph node metastasis, TNM stage, the status of estrogen receptor (ER), progesterone receptor (PR), human epidermal growth factor receptor (HER2), and E-cadherin were collected and recorded. Pathological TNM stages were determined according to the staging manual designed jointly by the Union International Against Cancer and the American Joint Committee on Cancer.

In addition, the correlation between SCUBE3 protein level and patient’s age (21–40, 41–60, 61–80, and 81–100), node metastasis status (N0, N1, N2, and N3), individual cancer stage (stage 1, stage 2, stage 3, and stage 4), and subclasses (luminal, HER2 + , and triple-negative) were analyzed using the UALCAN database (http://ualcan.path.uab.edu/index.html), which is an effective online analysis method, which uses The Cancer Genome Atlas (TCGA) levels 3 RNA-seq and clinical data from 31 cancer types to analyze gene expression data in depth [21]. We further supplemented and identified the SCUBE3 expression levels based on various classification parameters, such as the ER (ER + /ER−), PR (PR + /PR−), HER2 status (HER2 + /HER2−), and nodal status (N + /N−) using bc-GenExMiner v4.4 (http://bcgenex.centregauducheau.fr/) from TCGA data. (n = 1 034) [22].

### SCUBE3 expression and the prognosis in breast cancer patients

Overall survival (OS) is defined as the time from primary diagnosis to death resulting. Relapse-free survival (RFS) is defined as the time from primary diagnosis to recurrence all types of disease. To identify the correlation between SCUBE3 expression and clinical characteristics of breast cancer patients, we investigated the relationship between SCUBE3 expression and prognosis of breast cancer in TMN (TMN1, TMN2, and TMN3), tumor size (T ≤ 2 cm and T ≥ 2 cm), lymph node ( ±), histologic grade (grade 2 and grade 3), histologic type (invasive ductal and other types), ER (ER + /ER−), PR (PR + /PR−), HER2 status (HER2 + /HER2−), using the Kaplan–Meier survival curves based on HR and log-rank p-values [23].

### Statistical analysis

We first compared the data distribution of each covariate between the different staining groups, using *t* test (normal distribution) or Kruskal–Wallis rank-sum test (non-normal distribution) for continuous variables (presented as mean ± SD) and χ^2^ tests (non-normal distribution) for categorical data (Table 1). Secondly, OS and RFS probability distributions were studied using the Kaplan–Meier analysis, and the equality of survival curves was tested using the log-rank test. Then, univariate Cox regression (Table 2) was used to estimate the hazard ratio (HR) and 95% confidence intervals (CIs) to identify significant prognostic factors. The independent effect of SCUBE3 expression was examined after adjusting for other variables (Table 3) in a multivariate Cox regression model. Finally, the stratified analyses and interaction analyses were used to estimate the HR and 95% CIs to investigate the risk factors in every subgroup and the interaction terms of other factors, and Kaplan–Meier curves of cumulative hazards were stratified by SCUBE3 categories in different subgroups. HR for 95% confidence interval and p < 0.05 were considered statistically significant. All data were double entered and then exported to tab-delimited text files. Data were analyzed using the statistical packages R (version 3.5.3) and EmpowerStats (www.empowerstats.com; X&Y Solutions, Inc.).

**Table 2:** Effects of death risk factors on breast cancer by univariate analysis (N = 137)

Variable	Total	HR (95% CI)	P-value
SCUBE3			
Low	106 (77.4%)	Reference	
High	31 (22.6%)	2.77 (1.32, 5.80)	0.0070
Age, years	55.89 ± 11.73	1.05 (1.02, 1.08)	0.0025
Histologic grade			
2	95 (69.3%)	Reference	
3	42 (30.7%)	2.14 (1.03, 4.44)	0.0422
Histologic type			
Invasive ductal	114 (83.2%)	Reference	
Other	23 (16.8%)	1.24(0.50, 3.03)	0.6445
Tumor size, cm			0.2435
≤ 2	55 (40.1%)	Reference	
> 2	82 (59.9%)	1.28 (0.56, 2.74)	0.5329
Lymph node metastasis			
Negative	72 (52.6%)	Reference	
Positive	65 (47.4%)	2.73 (1.24, 6.00)	0.0123
TMN stage			
I	39 (28.5%)	Reference	
II	52 (37.9%)	2.45 (0.66, 9.05)	0.1789
III	46 (33.6%)	5.70 (1.67, 19.46)	0.0055
ER			
Negative	41 (30.6%)	Reference	
Positive	93 (69.4%)	0.32 (0.15, 0.67)	0.0023
Missing	2	1	1
PR			
Negative	45 (33.1%)	Reference	
Positive	91 (66.9%)	0.73 (0.35, 1.51)	0.0395
Missing	1	-	1
HER2			
Negative	100 (80.6%)	Reference	
Positive	24 (19.4%)	1.22 (0.49, 3.04)	0.6676
Missing	13	11	2
E-cadherin			
Negative	45 (38.1%)	Reference	
Positive	73 (61.9%)	0.9 (0.43, 1.89)	0.7886
Missing	19	14	5
TNBC			
No	108 (82.4%)	Reference	
Yes	23 (17.6%)	2.61 (1.17, 5.81)	0.0189
Missing	6	4	2

*ER* estrogen receptor, *PR* progesterone receptor, *HER2* human epidermal growth factor receptor

TMN stage was according to the seventh edition of the Guidelines for the American Journal of Critical Care. Variables are expressed as n (%)

**Table 3:** Multivariate COX regression for SCUBE3 and breast cancer mortality risk

SCUBE3	Non-adjusted	P-value	Adjust I	P-value	Adjust II	P-value
Low	Reference		Reference		Reference	
High	2.77 (1.32,5.80)	0.0070	3.28 (1.54,6.98)	0.0020	2.80 (1.20,6.51)	0.0168
Per 1 point increase	1.69 (1.06,2.70)	0.0278	1.88 (1.17,3.02)	0.0092	1.86 (1.06,3.25)	0.0300

Non-adjusted model adjusted for: None

Adjust I model adjusted for: Age

Adjust II model adjusted for: Age; Histologic grade; Histologic type; Tumor size; HER2; E-cadherin; ER

Per 1 point increase: Per 1 point for SCUBE immunohistochemical staining intensity

## Results

### SCUBE3 expression in different cancers

**Fig. 1:**
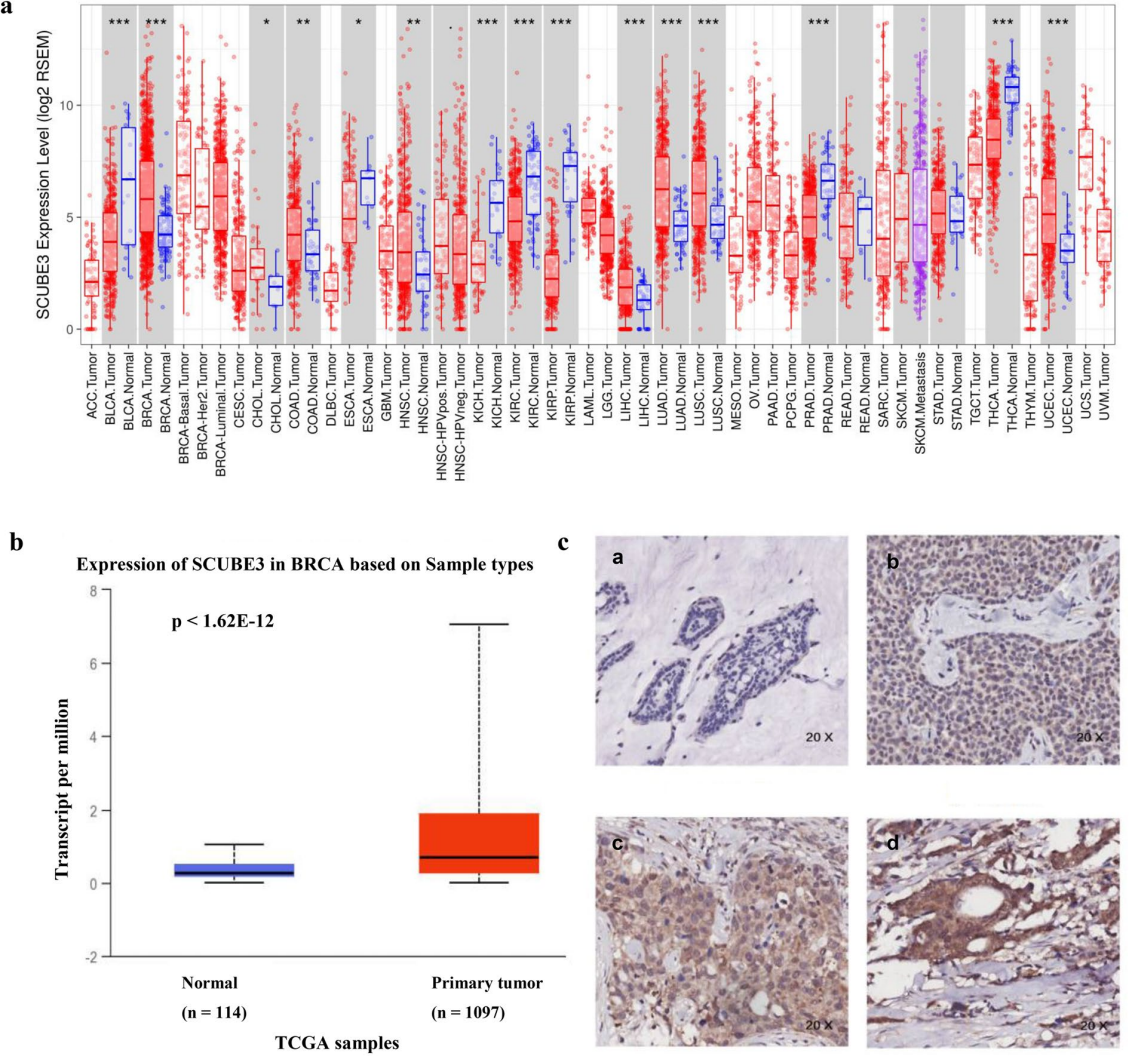
The expression of SCUBE3 in different cancers. **(a)** Human SCUBE3 expression levels in different tumor types from the TCGA database were determined by TIMER. **(b)** Expression of SCUBE3 in BRCA based on sample types, primary tumor (n = 1 097), normal (114), p < 1.62E-12. **(c)** Immunohistochemical staining of SCUBE3 protein in 137 tissues. Original magnification, 20 × . a Negative SCUBE3 expression (−) in breast cancer tissue samples. b Weak SCUBE3 expression ( +) in breast cancer tissue samples. c Moderate SCUBE3 expression (+ +) in breast cancer tissue samples. d Strong SCUBE3 expression (+ + +) in BRCA tissue samples

The expression of SCUBE3 in multiple cancer types (n = 32) was analyzed by the TIMER database. The differential expression of SCUBE3 between the tumor and adjacent normal tissues in all TCGA tumors were shown in Fig. 1a, in which SCUBE3 was overexpressed in breast cancer, but with relatively low expression in bladder urothelial carcinoma, kidney chromophobe, kidney renal clear cell carcinoma, kidney renal papillary cell carcinoma, prostate adenocarcinoma, and thyroid carcinoma. These results suggest that the transcription level of SCUBE3 is cancer type-specific.

To further determine the expression of SCUBE3, the relationship between SCUBE3 expression and sample type (normal tissue/primary breast cancer tumor) was further analyzed by the UALCAN database. As shown in Fig. 1b, SCUBE3 transcription levels in primary tumor tissue were significantly higher than normal subjects (p ≤ 1.62E-12). Furthermore, the representative immunohistochemical staining patterns for SCUBE3 were analyzed, SCUBE3 staining was primarily seen in the cytoplasm of cancer cells, while nuclear staining was also observed in ~ 30% of cancer cells (Fig. 1c). Of note, almost all tumor cells (~ 95%) were positively stained with SCUBE3 though the staining intensity varied. The expression levels of SCUBE3 were quantitated by scoring staining intensity, including negative (–), weak ( +), moderate (+ +), and strong (+ + +) staining, respectively. The results of SCUBE3 immunostaining were further classified into two groups, i.e., the low expression group (−, +) and the high expression group (+ + , +  + +). Among the 137 tumor samples, 106 samples showed low expression (77.3%), and the remaining 31 samples showed high expression (22.7%). Together, these results demonstrate that SCUBE3 is differentially expressed in breast cancer patients.

### Protein interaction and enrichment analysis of SCUBE3

**Fig. 2:**
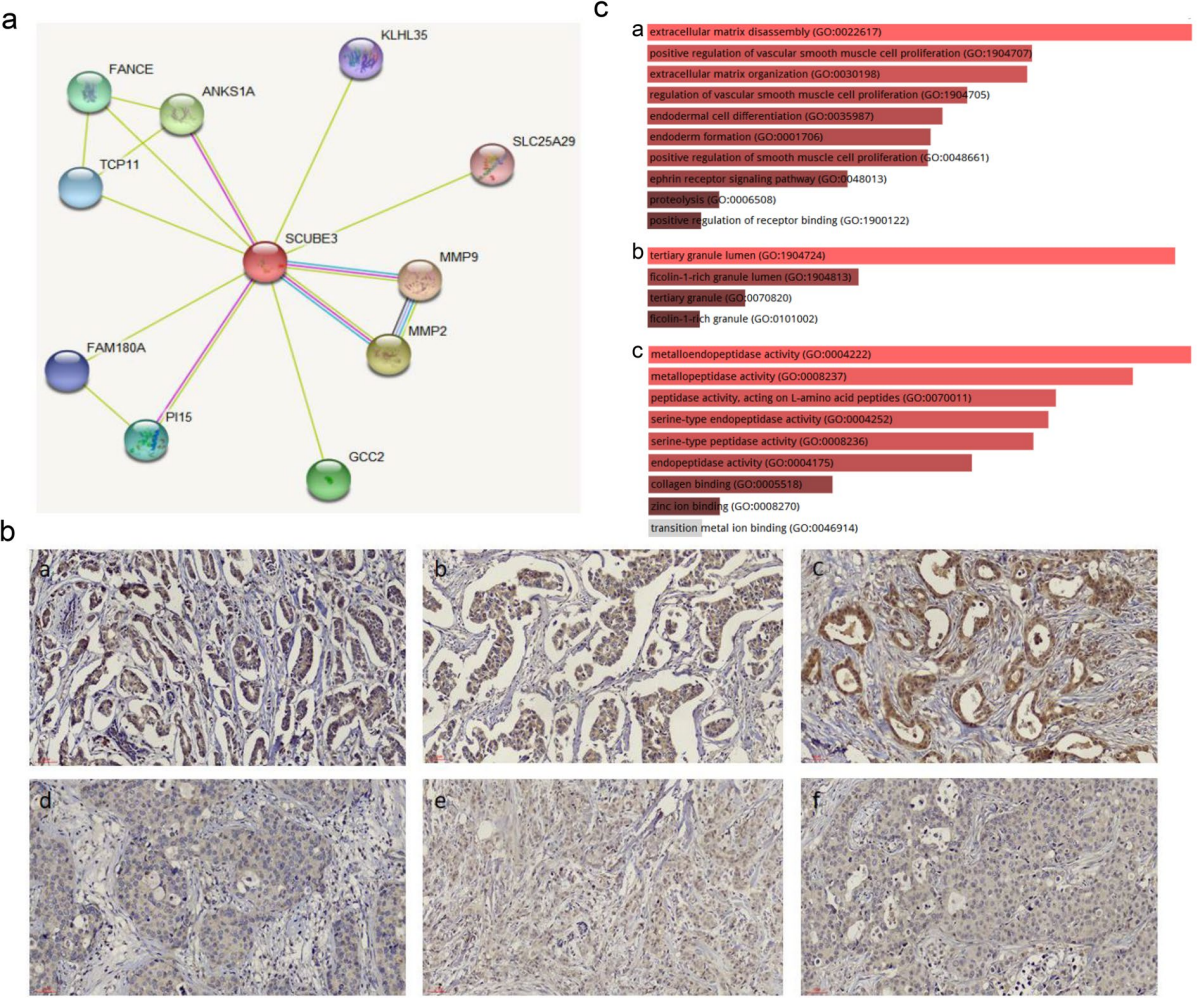
Protein interaction and enrichment analysis of SCUBE3. **(a)** Identification of known and predicted structural proteins essential for SCUBE3 function (STRING). Interacting proteins are displayed in colored circles using STRING. Predicted functional partners of SCUBE3 are shown based upon the peer-reviewed published data and curated database entries. **(b)** The relationship between SCUBE3 and MMP by immunohistochemistry. **(c)** Significantly enriched GO annotations of SCUBE3, MMP2, and MMP9. The bubble diagrams display the enrichment results of SCUBE3, MMP2, and MMP9. a biological processes; b cellular components; c molecular functions

STRING was applied to determine the proteins interacting with SCUBE3, and the results were shown in Fig. 2a. The following ten proteins were found to interact with SCUBE3, matrix metalloproteinase-9 (MMP-9), MMP-2, ankyrin repeat and SAM domain-containing protein 1A (ANKS1A), GRIP and coiled-coil domain-containing protein 2 (GCC2), fanconi anemia group E protein (FANCE), peptidase inhibitor 15 (PI15), T-complex protein 11 homolog (TCP11), family with sequence similarity 180 member A (FAM180A), kelch-like protein 35 (KLHL35) and solute carrier family 25 member 29 (SLC25A29), and their correlation scores were 0.934, 0.930, 0.682, 0.677, 0.663, 0.580, 0.577, 0.563, 0.542, and 0.541, respectively. Among these proteins, MMP-2 and MMP-9 showed the highest correlation with SCUBE3, suggesting that they may be functional partners in breast cancer. Previous studies have shown that MMPs could promote tumor invasion, metastasis, and angiogenesis after SCUBE3 was knocked out [6]. Especially, MMP2 and MMP9 are of particular interest for their role in the development and progression of early cancer [24]. Interestingly, we found that the above two proteins, MMP2 and MMP9 had the highest correlation with SCUBE3. We verified the functional relationship between SCUBE3 and MMP by IHC. Interestingly, we found that the expression levels of MMP2 and MMP9 were higher in samples with SCUBE3 strong staining (Fig. 2B a–c), while the expression levels of MMP2 and MMP9 were relatively low in samples with SCUBE3 weak staining (Fig. 2B d–f), indicating that the expression of SCUBE3 was positively correlated with the expression of MMP2 and MMP9 in breast cancer, suggesting that SCUBE3 may be closely related to the development and progression of cancer.

To further understand the biological functions of the above proteins, we performed GO enrichment analysis. As shown in Fig. 2C a, these proteins play a major role in the positive regulation of vascular smooth muscle cell proliferation, keratinocyte migration, and receptor binding, while cellular components included tertiary granule lumen and ficolin-1-rich granule lumen (Fig. 2C b). Molecular function analysis showed that the enriched GO terms were metalloendopeptidase activity, metallopeptidase activity, collagen binding, peptidase activity, acting on L-amino acid peptides, and serine-type endopeptidase activity (Fig. 2C c). Previous studies have shown that metallopeptidase has a relationship with cancer cell migration and invasion [25, 26]. Collagen is the main component of the extracellular matrix and is highly expressed in a variety of tumors. It was hypothesized a promising target for cancer treatment because it affects the microenvironment of tumors by increasing the recruitment of macrophages cells [27]. Therefore, we speculate that SCUBE3 may be related to the occurrence and development of cancer.

### SCUBE3 expression and clinical-pathological parameters of patients with breast cancer

To better understand the relevance and underlying mechanisms of SCUBE3 expression in breast cancer, we summarized the distribution of clinicopathological information of patients in SCUBE3 high expression group and SCUBE3 low expression group. Of the 137 breast cancer patients, 31 cases (22.6%) were identified as high SCUBE3 expression (Table 1). According to the expression of SCUBE3, the study population was divided into two groups: low expression group and high expression group. The data showed that the distribution of breast cancer patients in the high expression group and the low expression group was significantly different in ER, PR, HER2, E-cadherin, and survival state (p < 0.05), but there was no significant difference in histologic grade, histologic type, tumor size, lymph node metastasis, TMN stage, subtypes, or recurrence (p > 0.05). Positive ER expression was associated with low SCUBE3 expression, positive PR expression was associated with low SCUBE3 expression.

**Fig. 3:**
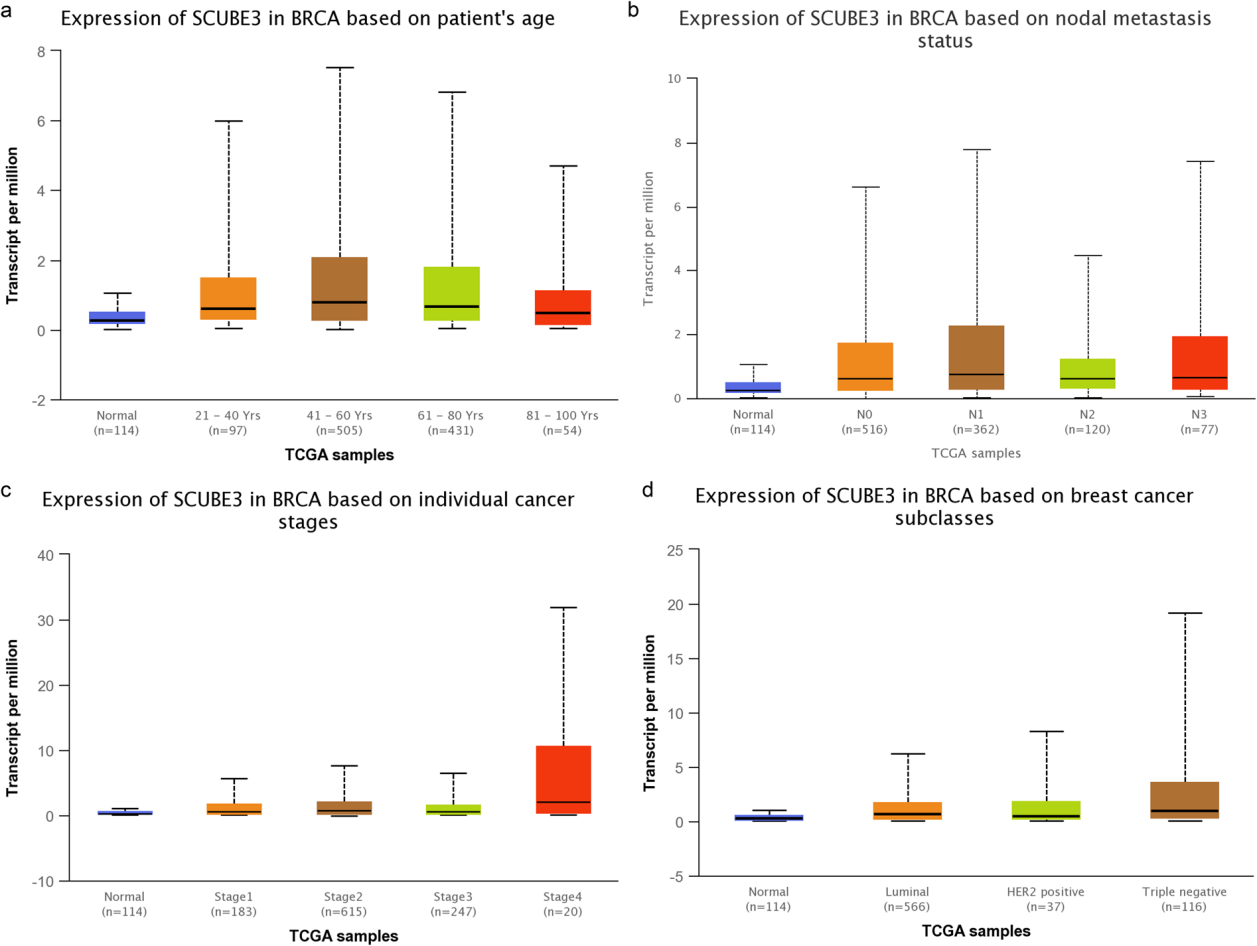
UALCAN analysis for the correlation between SCUBE3 mRNA expression level and clinicopathological parameters of breast cancer. (**a**) patient’s age (21–40, 41–60, 61–80, and 81–100), (**b**) node metastasis status (N0, N1, N2, and N3), (**c**) individual cancer stage (stage 1, stage 2, stage 3, and stage 4), (**d**) subclasses (luminal, HER2 + , and triple-negative)

We further examined the expression of SCUBE3 in breast cancer based on patient’s age (21–40, 41–60, 61–80, and 81–100), node metastasis status (N0, N1, N2, and N3), individual cancer stage (stage 1, stage 2, stage 3, and stage 4), and subclasses (luminal, HER2 + , and triple negative) using the UALCAN database. As shown in Fig. 3 and Additional file 1: Table, the expression of SCUBE3 in patients with Y21-40, Y41-60, Y61-80, and Y81-100 had significantly higher than that in normal tissues (Normal-vs-Age (21–40 Yrs), p = 1.23E-04; Normal-vs-Age (41–60 Yrs), p = 3.15E-12; Normal-vs-Age (61–80 Yrs), p = 3.88E-09; Normal-vs-Age (81–100 Yrs), p = 2.68E-03), However, there was no significant difference in the expression of SCUBE3 among Y21-40, Y41-60, Y61-80, and Y81-100. In comparing node metastasis status, we found that SCUBE3 expression was higher in N0, N1, N2, and N3 than that in the normal group (Normal-vs-N0, p = 8.63E-11; Normal-vs-N1, p = 6.86E-11; Normal-vs-N2, p = 3.02E-03; Normal-vs-N3, p = 1.58E-05), but there was no significant difference among N0, N1, N2, and N3. An analysis of individual cancer status showed that SCUBE3 expression in stage 2 was significantly higher than that in stage 1 (p = 2.87E-02), and that in stage 3 was significantly higher than that in stage 2 (p = 1.26E-02). Besides, we analyzed the expression of SCUBE3 based on subtypes, the results showed that the expression of SCUBE3 in different subtypes than that in normal breast tissues (Normal-vs-Luminal, p = 4.17E-12; Normal-vs-HER2 Positive, p = 4.07E-02; Normal-vs-TNBC, p = 1.17E-04). The expression of SCUBE3 was higher in TNBC than in luminal and HER2 + (Luminal-vs-TNBC, p = 8.56E-03; HER2 Positive-vs-TNBC, p = 2.46E-02).

**Fig. 4:**
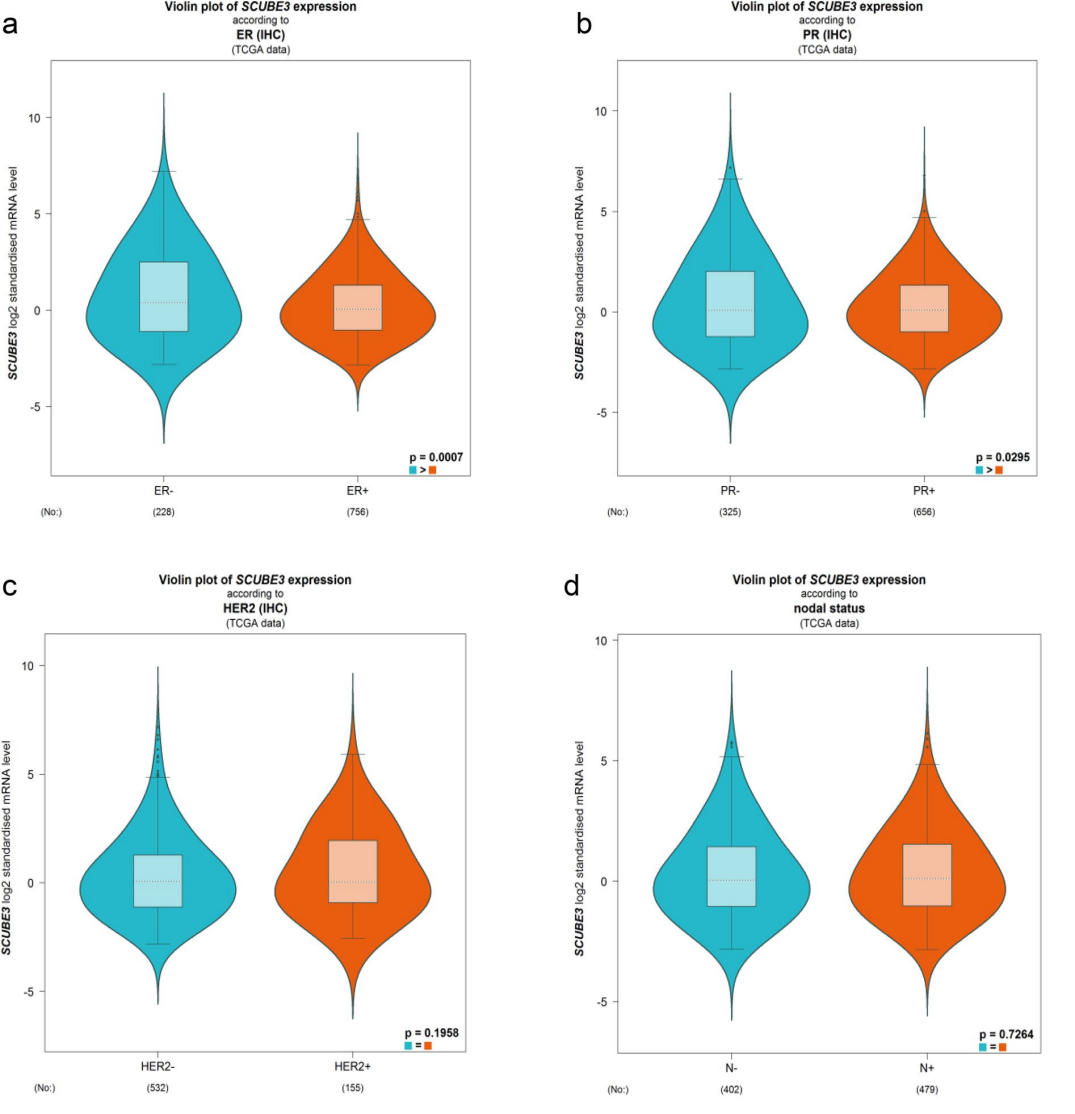
The relationship between SUBE3 expression and clinical-pathological parameters by bc-GenExMiner v4.4. **a** ER (ER + /ER-), **b** PR (PR + /PR-), **c** HER2 status (HER2 + /HER2 −), **d** nodal status (N + /N −)

We supplemented SCUBE3 expression and clinical-pathological parameters based on ER (ER + /ER-), PR (PR + /PR-), HER2 status (HER2 + /HER2-), and nodal status (N + /N-) using bc-GenExMiner v4.4 (Fig. 4). The results showed that there were remarkably different expression levels of SCUBE3 mRNA in ER status (ER- > ER + , p = 0.0007), and PR status (PR- > PR + , p = 0.0295), respectively. However, there was no significant expression difference of SCUBE3 mRNA in HER2 receptor status and nodal status (p > 0.05). Taken together, SCUBE3 expression was significantly different in ER, and PR, suggesting that SCUBE3 expression may serve as a potential diagnostic indicator in breast cancer.

### High SCUBE3 expression correlates with poor prognosis in breast cancer

**Fig. 5:**
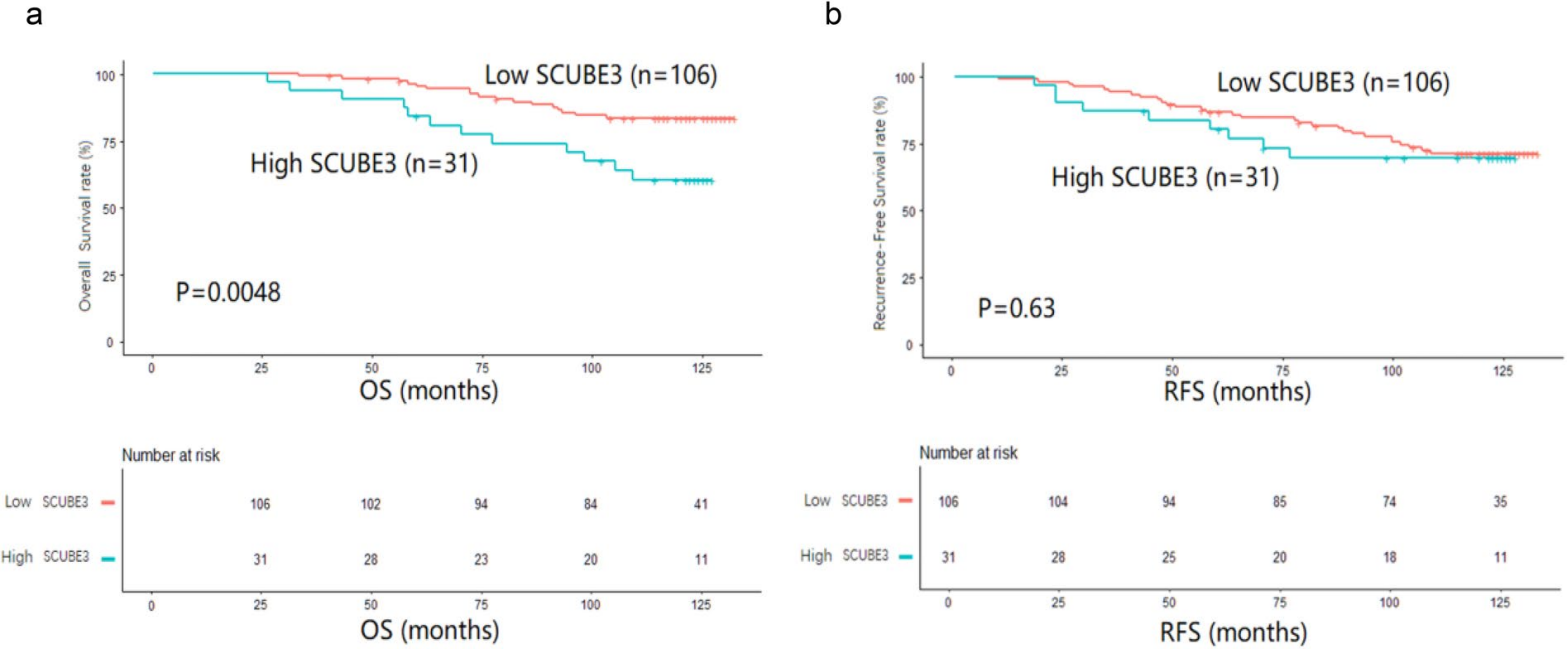
The relationship between SCUBE3 expression and prognosis of breast cancer. High SCUBE3 expression was associated with poor prognosis (**a**: OS: p = 0.0048;** b**: RFS: p = 0.63)

To further examine the prognostic potential of SCUBE3 in breast cancer, we then analyzed the impact of SCUBE3 expression on the prognosis of breast cancer patients. The average time was 108 months (low expression group, 89 months versus; high expression group, 19 months). Of note, the high expression of SCUBE3 was associated with poor prognosis (p < 0.05) (Table 1). Similar results were observed for Kaplan–Meier analysis (Fig. 5), in which the OS and RFS rates in the high vs. low SCUBE3 expression groups were 38.7% vs. 16.0% (p = 0.0048) and 29.0% vs. 27.4% (p = 0.63), respectively. In addition, the cumulative recurrence risk was significantly increased in the high SCUBE3 expression group. These data suggest that high SCUBE3 expression is correlated with poorer prognosis in breast cancer.

### SCUBE3 is an independent prognostic factor in breast cancer

**Fig. 6:**
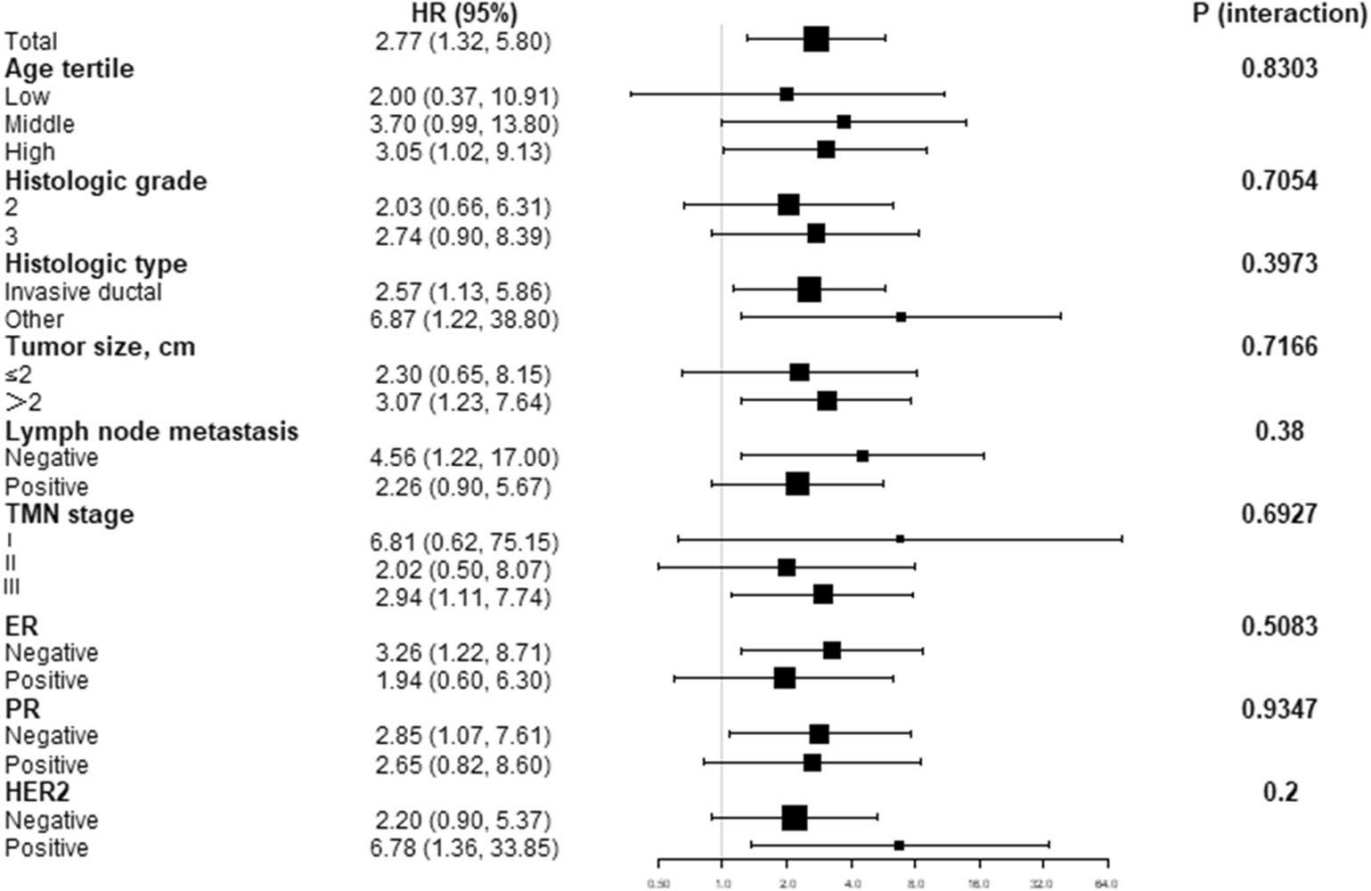
The stratified analysis and interaction test of the independent predictive effect of SCUBE3 expression on the risk of death in patients from Taizhou Hospital in Zhejiang Province has a stable effect in each subgroup (p > 0.05)

We further analyzed the effects of death risk factors on breast cancer by univariate Cox regression analysis. As shown in Table 2, high SCUBE3 expression is associated with a significantly increased risk of death for breast cancer patients (HR: 2.77, 95% CI 1.32–5.80, p = 0.0070). In addition to SCUBE3 expression, significant factors associated with the risk of death were patient age (p = 0.0025), histologic grade (p = 0.0422), lymph node metastasis (p = 0.0123), TMN stage III (p = 0.0055), ER expression status (p = 0.0023), and TNBC subtype (p = 0.0189). To demonstrate whether SCUBE3 expression is an independent prognostic factor for BRCA, multivariate Cox regression analysis was performed after adjusting potential confounding factors, including age, histologic grade, histologic type, tumor size, HER2, E-cadherin, and ER. As shown in Table 3, the HR was 2.80 (95% CI 1.20–6.51, p = 0.0168) in individuals with high SCUBE3 expression, and HR was increased by 1.86 (95% CI: 1.06–3.25, p = 0.0300) for per 1-point increase of SCUBE3 expression. Finally, the stratified analyses and interaction analyses were used to estimate the HRs and 95% CIs to investigate the risk factors in every subgroup and the interaction terms of other factors (Fig. 6). The results showed that the independent predictive effect of the expression of SCUBE3 in patients on the risk of death was stable in each subgroup (p > 0.05).

**Fig. 7:**
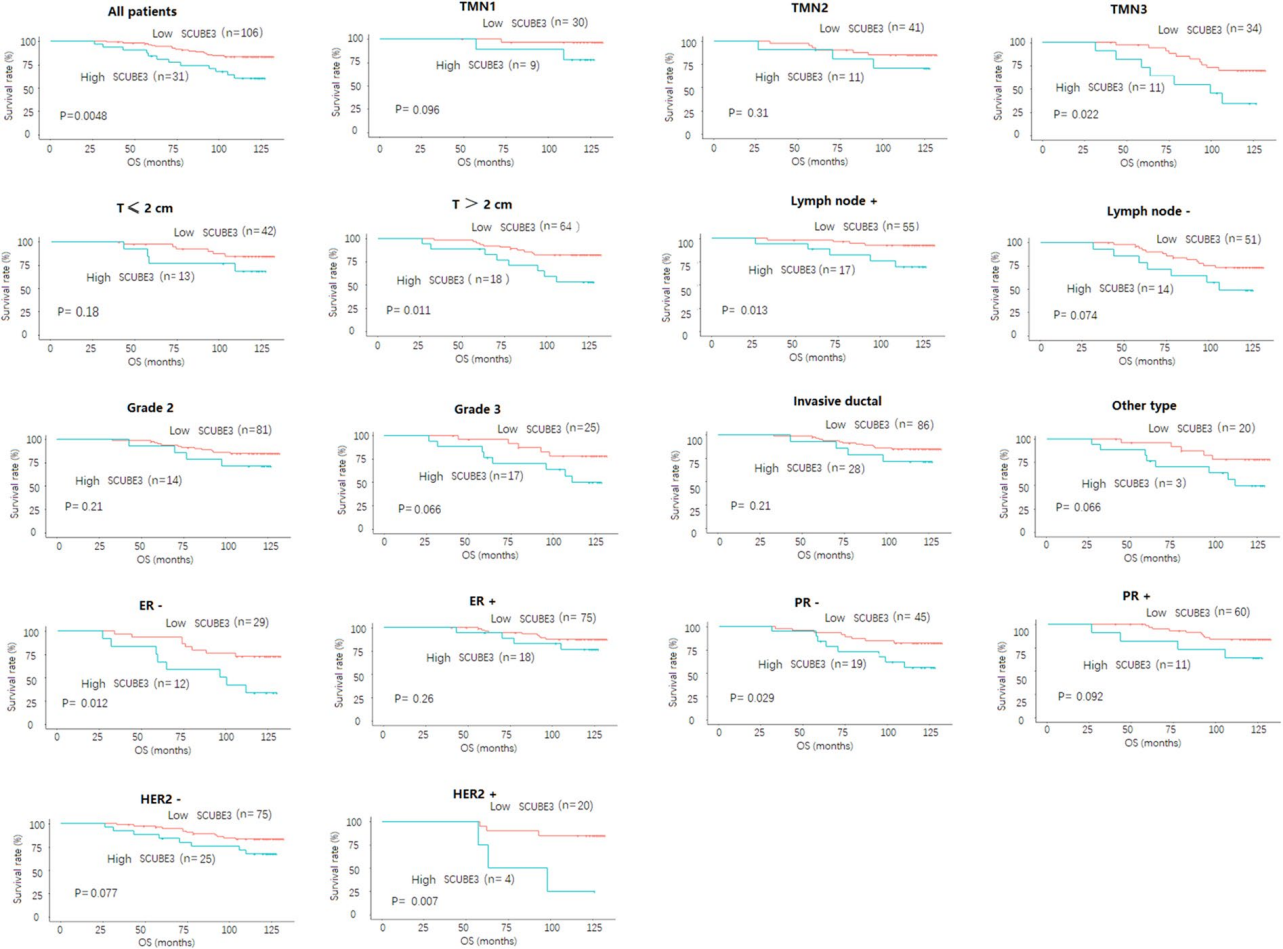
The impact of SCUBE3 expression on prognosis of breast cancer patients in each subgroup. All patients; TMN (TMN1, TMN2, and TMN3); tumor size (T ≤ 2 cm and T ≥ 2 cm); lymph node ( ±); histologic grade (grade 2 and grade 3); histologic type (invasive ductal and other types); ER (ER + /ER-); PR (PR + /PR-); HER2 status (HER2 + /HER2-); lymph node status (positive and negative); and grade (grade 2 and grade 3) using the Kaplan–Meier survival curves based on the hazard ratios (HR) and log-rank p-values

Lastly, we divided our study population into different subgroups based on TMN stage (TMN1, TMN2, and TMN3), tumor size (T ≤ 2 cm and T ≥ 2 cm), lymph node ( ±), histologic grade (grade 2 and grade 3), histologic type (invasive ductal and other types), ER (ER + /ER−), PR (PR + /PR−), and HER2 (HER2 + /HER2−), and then analyzed the impact of SCUBE3 expression on prognosis of breast cancer patients in each subgroup. As shown in Fig. 7, the Kaplan–Meier survival curve showed the survival of patients with high SCUBE3 expression was persistently consistently worse than that with low SCUBE3 expression in each subgroup (TMN3, p = 0.022; T ≥ 2 cm, p = 0.011; lymph node ( +), p = 0.013; ER−, p = 0.012; PR−, p = 0.029; HER2 + , p = 0.007). Taken together, these findings indicate that SCUBE3 may be an independent prognostic indicator of mortality risk in patients with breast cancer.

## Discussion

The lack of effective and reliable prognostic biomarkers remains a major problem for improving the clinical outcomes of breast cancer patients. In this study, we aim to explore and evaluate the prognostic values of SCUBE3 expression in breast cancer using clinical data of these patients and extensive bioinformatics data mining process. We have comprehensively investigated the expression pattern of SCUBE3 in breast cancer and analyzed the proteins that interact with SCUBE3. Then, we systematically evaluated SCUBE3 expression in a cohort of breast carcinoma samples collected for postoperative pathological diagnosis in a retrospective study. Lastly, we analyzed the correlation of SCUBE3 expression and the prognosis of breast cancer, and demonstrated that SCUBE3 was an independent poor prognostic factor in breast cancer. Our data were consistent with most previous studies reported in other cancer types including NSCLC [14], osteosarcoma [28], and renal cell carcinoma [29], which suggested that high SCUBE3 expression may be a universal indicator of poorer prognosis in cancers (Additional file 2).

SCUBE3 was first identified in human umbilical vein endothelial cells [8]. It has been reported that purified SCUBE3 protein and C-terminal CUB domain fragment bind to TGF-β type II receptor through C-terminal CUB, activate TGF-β signal transduction, and trigger epithelial-mesenchymal transition EMT. This process includes the induction of Smad2/3 phosphorylation, the increase of Smad2/3 transcriptional activity, the expression of up-regulated Snail and Slug, thus promoting cancer cell migration and invasion. Besides, SCUBE3 has been reported to be involved in angiogenesis [11, 25].

We examined the expression levels of SCUBE3 in breast cancer using TIMER, UALCAN, and immunohistochemistry. We found that SCUBE3 was highly expressed in breast cancer. In addition, the protein–protein interaction and functional enrichment data demonstrated that the gelatinases MMP-2 and MMP-9 showed the highest correlation with SCUBE3. It is well known that MMPs promote tumor invasion, metastasis, and angiogenesis via degrading type IV collagen in the basal layer of blood vessels, initiating integrin signaling and lysing ECM-bound pro-angiogenic factors [26, 27, 30]. In particular, MMP-2/9 overexpression has been reported to be a poor prognostic factor for breast cancer [31]. Therefore, the close interaction between SCUBE3 and MMP-2/9 may suggest the role of SCUBE3 in the metastasis and progression of breast cancer, though future mechanistic studies are needed to verify this possibility. To explore the potential mechanism of SCUBE3 in breast cancer, we analyzed the functional protein association network. Our study found that the molecular function of SCUBE3 was mainly enhanced in metalloendopeptidase activity, metallopeptidase activity, collagen binding, peptidase activity, acting on L-amino acid peptides, and serine-type endopeptidase activity. These results reveal the potential regulating role of SCUBE3 in breast cancer. As is known to high-grade breast cancer usually displays relatively aggressive clinical behavior and indolent clinical course. Their natural history, molecular features, and therapeutic strategies are also significantly different from those of low-grade breast cancer [32]. Besides tumor grade, E-cadherin inactivation also critically contributes to the development and progression of distinct types in the breast cancer [28]. Liang W et al. revealed that SCUBE3 was closely related to the prognosis of the patients, suggesting that SCUBE3 can inhibit the tumor proliferation and may become a new therapeutic target [33]. Our data suggest that high SCUBE3 expression is correlated with high histologic grade and negative E-cadherin expression, which further support the important role of SCUBE3 in promoting breast cancer progression as well as its relationship with tumor metastasis and poor prognosis. These findings provide an important possibility for using SCUBE3 expression as a biomarker to identify breast cancer patients who are more likely to have poor prognosis and to help design more effective treatment and follow-up plans for these patients.

Another important aspect of this study is that SCUBE3 expression is related to the clinical-pathological parameters of patients with breast cancer. We found that the expression of SCUBE3 was associated with cancer stage and different subtypes. Compared with the early stage, the expression of SCUBE3 is higher in late-stage cancers, which indicates that SCUBE3 may play a role in cancer progression and invasion. The expression of SCUBE3 was higher in TNBC than in luminal, HER2 + and TNBC subtypes. Also, we found that there were remarkably different expression levels of SCUBE3 mRNA in ER and PR status. However, there was no significant difference in the expression of SCUBE3 in age, HER2 receptor status, and nodal status (p > 0.05). These results suggest that SCUBE3 expression may serve as a potential diagnostic indicator in breast cancer.

To investigate whether the survival rate of breast cancer patients is related to the expression level of SCUBE3, we have investigated the correlation between SCUBE3 expression and survival rate of breast cancer patients using Kaplan–Meier analysis. We analyzed the correlation between high expression of SCUBE3 and poor survival rate by multivariate Cox regression analysis, and proved that high expression of SCUBE3 can be used as an independent prognostic factor for breast cancer patients. Furthermore, we performed stratified analysis and interaction analysis to ensure the correlation between high SCUBE3 expression and poor prognosis existing based on OS and RFS. Indeed, the survival status of high SCUBE3 expression group is persistently worse than that of low SCUBE3 expression group, which further supports the high SCUBE3 expression as an independent factor for the poor prognosis of breast cancer patients. Kaplan–Meier analysis showed that the high SCUBE3 expression was associated with poor prognosis in TMN3, lymph node ( +), ER−, PR-, and HER2 + . Taken together, these findings suggest high expression of SCUBE3 in breast cancer was associated with poor prognosis and may be a prognostic indicator of mortality risk in breast cancer patients.

There are a few limitations to this retrospective study. Firstly, the data from the database do not provide sufficient information about the protein level and activities of SCUBE3. Secondly, although there is a clear association between SCUBE3 expression and the prognosis of breast patients, the sample size (137 patients) was relatively small. Therefore, our conclusion should be validated in more clinical samples and large-scale studies.

In summary, our study explored the role of SCUBE3 in breast cancer by using extensive bioinformatics data, and demonstrated that high expression of SCUBE3 is associated with poor prognosis in breast cancer patients, and may serve as an independent poor prognostic factor and a potential therapeutic target for breast cancer.

## Supplementary Information

**Additional file 1: Table S1.** The correlation between SCUBE3 expression levels and clinical-pathological parameters.

**Additional file 2.** The original data.

## Data Availability

The data underlying this study are freely available from TIMER (https://cistrome.shinyapps.io/timer/), the UALCAN database (http://ualcan.path.uab.edu/index.html), bc-GenExMiner v4.4 (http://bcgenex.centregauducheau.fr/), the STRING database (https://string-db.org/), and the Enrichr (http://amp.pharm.mssm.edu/Enrichr).
