# Supplementary material for: SCUBE3 serves as an independent poor prognostic factor in breast cancer
Source: Cancer Cell Int. 2021 May 18;21:268. doi: 10.1186/s12935-021-01947-3 (PMC8130162; doi:10.1186/s12935-021-01947-3)
Supplement: Supplementary file 1 — Additional file 1: Table S1. The correlation between SCUBE3 expression levels and clinical-pathological parameters. [file 12935_2021_1947_MOESM1_ESM.doc]

|  | **Comparison** | **Statistical significance** |
| --- | --- | --- |
| Patient’s age | **Normal-vs-Age(21-40Yrs)** | **1.226520E-04** |
|  | **Normal-vs-Age(41-60Yrs)** | **3.14792636402217E-12** |
|  | **Normal-vs-Age(61-80Yrs)** | **3.87520004974107E-09** |
|  | **Normal-vs-Age(81-100Yrs)** | **2.684200E-03** |
|  | Age(21-40Yrs)-vs-Age(41-60Yrs) | 7.361800E-01 |
|  | Age(21-40Yrs)-vs-Age(61-80Yrs) | 6.224600E-01 |
|  | Age(21-40Yrs)-vs-Age(81-100Yrs) | 3.560600E-01 |
|  | Age(41-60Yrs)-vs-Age(61-80Yrs) | 8.188400E-01 |
|  | Age(41-60Yrs)-vs-Age(81-100Yrs) | 1.325200E-01 |
|  | Age(61-80Yrs)-vs-Age(81-100Yrs) | 1.162220E-01 |
| Node metastasis status | **Normal-vs-N0** | **8.62789839572997E-11** |
|  | **Normal-vs-N1** | **6.85850265469412E-11** |
|  | **Normal-vs-N2** | **3.021900E-03** |
|  | **Normal-vs-N3** | **1.57591999999651E-05** |
|  | N0-vs-N1 | 1.933160E-01 |
|  | N0-vs-N2 | 1.170570E-01 |
|  | N0-vs-N3 | 1.574960E-01 |
|  | N1-vs-N2 | 5.159000E-01 |
|  | N1-vs-N3 | 7.708600E-01 |
|  | N2-vs-N3 | 7.074000E-01 |
| Individual cancer stage | **Normal-vs-Stage1** | **3.55080000000729E-05** |
|  | **Normal-vs-Stage2** | **2.00806038463952E-12** |
|  | **Normal-vs-Stage3** | **5.55890000697445E-10** |
|  | **Normal-vs-Stage4** | **2.802100E-02** |
|  | **Stage1-vs-Stage2** | **2.865700E-02** |
|  | Stage1-vs-Stage3 | 9.502400E-01 |
|  | Stage1-vs-Stage4 | 8.530500E-02 |
|  | **Stage2-vs-Stage3** | **1.263900E-02** |
|  | Stage2-vs-Stage4 | 1.714350E-01 |
|  | Stage3-vs-Stage4 | 8.559500E-02 |
| Subclasses | **Normal-vs-Luminal** | **4.16744416753545E-12** |
|  | **Normal-vs-HER2 Positive** | **4.072400E-02** |
|  | **Normal-vs-TNBC** | **1.166690E-04** |
|  | Luminal-vs-HER2 Positive | 9.560000E-01 |
|  | **Luminal-vs-TNBC** | **8.562200E-03** |
|  | **HER2 Positive-vs-TNBC** | **2.456300E-02** |

Table S1. The correlation between SCUBE3 expression levels and clinical-pathological parameters

Bold values indicate that p < 0.05
